# Targeted epigenome editing of an endogenous locus with chromatin modifiers is not stably maintained

**DOI:** 10.1186/s13072-015-0002-z

**Published:** 2015-03-18

**Authors:** Goran Kungulovski, Suneetha Nunna, Maria Thomas, Ulrich M Zanger, Richard Reinhardt, Albert Jeltsch

**Affiliations:** Institute of Biochemistry, Faculty of Chemistry, Stuttgart University, Pfaffenwaldring 55, 70569 Stuttgart, Germany; Dr. Margarete Fischer-Bosch Institute of Clinical Pharmacology, Auerbachstraße 112, 70376 Stuttgart, Germany; University of Tübingen, Geschwister-Scholl-Platz, 72074 Tübingen, Germany; Max-Planck-Genomzentrum Köln, Carl-von-Linné-Weg 10, 50829 Köln, Germany

## Abstract

**Background:**

DNA methylation and histone 3 lysine 9 (H3K9) methylation are considered as epigenetic marks that can be inherited through cell divisions. To explore the functional consequences and stability of these modifications, we employed targeted installment of DNA methylation and H3K9 methylation in the vascular endothelial growth factor A (VEGF-A) promoter using catalytic domains of DNA or H3K9 methyltransferases that are fused to a zinc finger protein which binds a site in the VEGF-A promoter.

**Results:**

Expression of the targeted DNA and H3K9 methyltransferases caused dense deposition of DNA methylation or H3K9 di- and trimethylation in the promoter of VEGF-A and downregulation of VEGF-A gene expression. We did not observe positive feedback between DNA methylation and H3K9 methylation. Upon loss of the targeted methyltransferases from the cells, the epigenetic marks, chromatin environment, and gene expression levels returned to their original state, indicating that both methylation marks were not stably propagated after their installment.

**Conclusions:**

The clear anti-correlation between DNA or H3K9 methylation and gene expression suggests a direct role of these marks in transcriptional control. The lack of maintenance of the transiently induced silenced chromatin state suggests that the stability of epigenetic signaling is based on an epigenetic network consisting of several molecular marks. Therefore, for stable reprogramming, either multivalent deposition of functionally related epigenetic marks or longer-lasting trigger stimuli might be necessary.

**Electronic supplementary material:**

The online version of this article (doi:10.1186/s13072-015-0002-z) contains supplementary material, which is available to authorized users.

## Background

Although (almost) all cell types in a multicellular organism contain the same genetic information, they are functionally and morphologically different. Such diversity can only be attained with highly regulated spatial and temporal control of gene expression enacted by the so-called epigenetic mechanisms. Epigenetics is currently understood by most researchers as a process for generation of heritable but reversible signals that do not alter the DNA sequence. These epigenetic mechanisms extend the information content of the genome and mediate the heritable propagation of transcriptional cellular programs and cellular identity, which is a cornerstone of organismic development [1-5]. Examples of inherited epigenetic signals [6] include imprinting and X-chromosome inactivation in mammals [7,8], and trans-generational inheritance of epigenetic states recently shown in plants [9] or worms [10]. Epigenetic signaling is not only essential for normal development but it plays a central role in the onset of diseases including cancer as well [11-14].

DNA methylation and post-translational modifications (PTMs) of histones are critical epigenetic signals. In mammalian cells, the DNA molecules can be modified by methylation at the 5 position of cytosines, typically in a CpG dinucleotide context, which in turn can be oxidized to 5-hydroxymethylcytosine and higher oxidation states [15]. In a similar vein, the n-terminal tails of histones can be massively modified, including methylation of lysine and arginine residues, acetylation of lysine, phosphorylation of serine and threonine, as well as ubiquitylation and sumoylation of lysines [4]. DNA methylation of CpG islands in gene promoters is correlated with gene repression, whilst DNA methylation of gene bodies is associated with gene expression [16,17]. Similarly, some histone modifications can be associated with silenced chromatin states (such as H3K9me3, H4K20me3, and H3K27me3) but others occur on actively transcribed chromatin (such as acetylation of histone H3 and H4, H3K4me2/3, and H3K36me3). The transmission of epigenetic marks over cell divisions can be explained by different molecular models: in the case of DNA methylation at palindromic CpG sites, the modification occurs on both strands of the DNA and after DNA replication, the hemimethylated sites can be specifically emethylated by DNA methyltransferase (Dnmt) 1 in a process of maintenance methylation [3,5]. For the maintenance of histone modifications, models propose a stochastic distribution of old modified histones on the two DNA daughter strands after replication and additional incorporation of new histones. Afterwards, the modification state can be copied from the old histones to the new ones by epigenetic ‘readers’, which bind a particular mark and then recruit a modifier, setting this mark. For example, in the case of H3K9 methylation, the HP1 protein binds di- and trimethylation lysine 9 moieties and then recruits the SUV39H1 and H2 enzymes, which can introduce H3K9me2 and H3K9me3 on nearby H3-tails [2,3].

The central role of epigenetic signaling in diseases, together with the principle reversibility of epigenetic states, has raised much interest in epigenetic editing approaches aiming to specifically modulate epigenetic states. One way to approach this is to use chimeric enzymes consisting of a targeting module that specifically binds defined DNA sequences (such as zinc fingers, transcription activator-like effectors (TALEs), or CRISPRs) and an effector domain that harbors a defined epigenetic activity, such as catalytic domains of DNA- or histone methyltransferases [18]. In the past years, a number of proof of principle studies have validated the feasibility of epigenetic editing in reporter plasmids, viral DNA, and endogenous targets (reviewed in [18]) and paved the way towards more comprehensive studies. These include zinc finger-targeted DNA methylation [19-22], TALE-targeted DNA demethylation [23,24], TALE-targeted H3K4me2 demethylation [25], or zinc finger-targeted H3K9 methylation [26,27]. However, the stability and heritability of such epigenetic editing remained an unsolved question.

**Figure 1:**
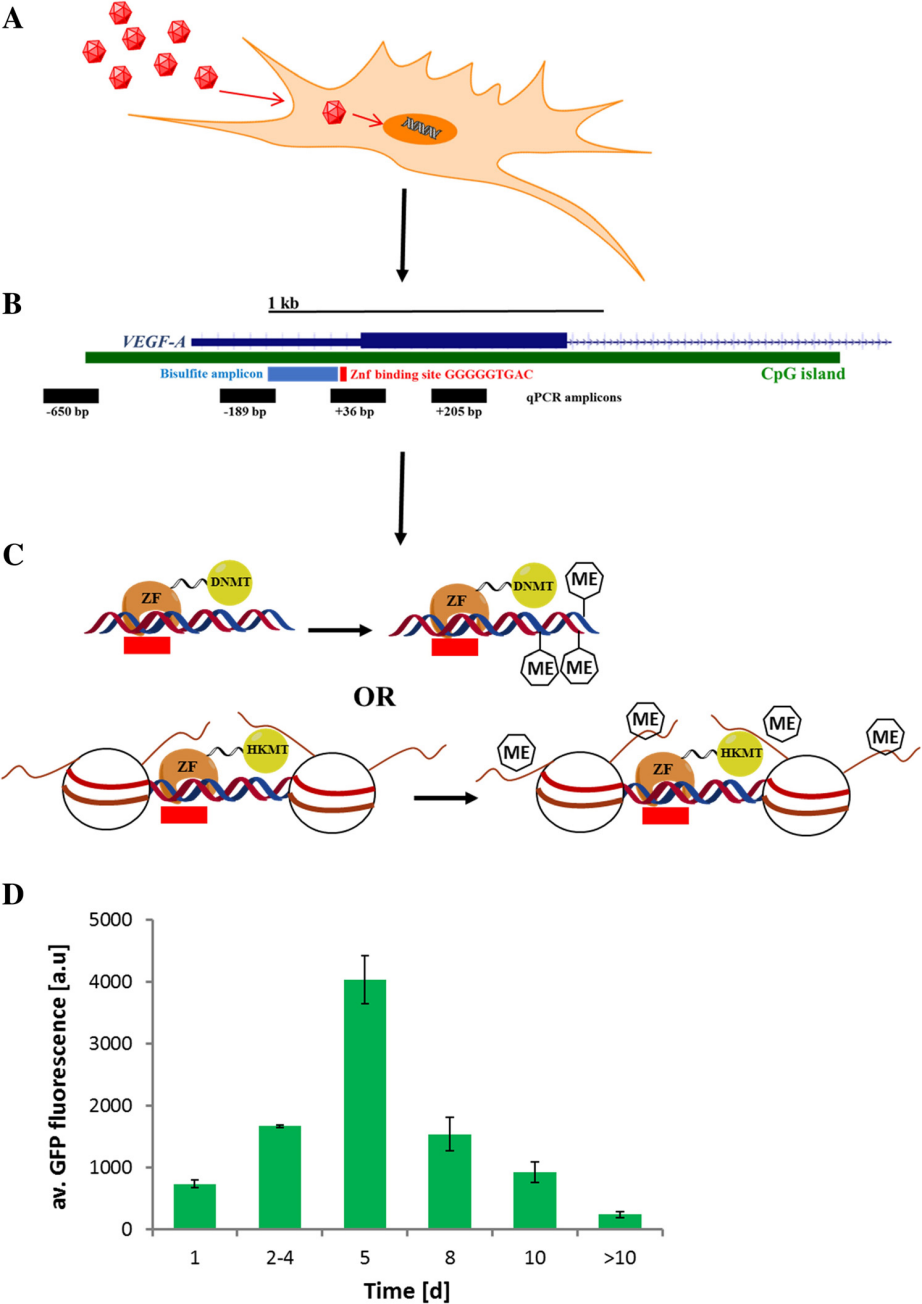
**Schematic overview of the strategy used in this study and time course of the expression of adenoviral-encoded genes. (A)** Adenoviral vectors harboring the genes of targeted DNA and H3K9 methyltransferases were used to infect SKOV3 cells. **(B)** The enzymes were fused to a zinc finger domain which binds a site in the promoter of the VEGF-A gene. The schematic picture of the VEGF-A promoter shows the gene structure in dark blue, an annotated CpG island in green, the zinc finger-binding site in red, and the amplicons used for bisulfite DNA methylation analysis and ChIP-qPCR in light blue and black, respectively. Additional ChIP-qPCR amplicons located outside of the region shown here are indicated in Figure 3. Transcription factor-binding sites to the VEGF-A promoter are indicated in Additional file 1: Figure S1A. **(C)** The chimeric zinc finger-fused DNA and H3K9 methyltransferases are targeted to the VEGF-A promoter where they introduce DNA or histone H3K9 methylation. **(D)** Time course of the expression of adenoviral vector-encoded genes after cell infection. The adenoviral vector also expresses GFP to allow for the FACS analysis of infection yields and follow the expression levels of virus-encoded genes. The time-course of GFP fluorescence of all data sets analysed in this respect was similar, and its average is shown here. The data shows averages and corresponding standard errors of the mean of 21 experiments. GFP, green fluorescence protein; ZF, zinc finger; HKMT, histone lysine methyltransferase; DNMT, DNA methyltransferase; d, days; bp, base pair; qPCR, quantitative PCR; VEGF-A, vascular endothelial growth factor A.

In this study, we have successfully designed and employed adenoviral vectors harboring a zinc finger protein binding to the vascular endothelial growth factor A (VEGF-A) gene promoter, fused via a flexible linker to the catalytic domain of a DNA or a histone H3K9 methyltransferase (Figure 1). Upon transient expression of the constructs in SKOV3 cells, we monitored the time-course of changes of DNA and H3K9 methylation, as well as VEGF-A gene expression and made intriguing observations. After infection with adenoviral vectors, we detected dense deposition of the epigenetic marks (DNA methylation or H3K9 di- and trimethylation), which peaked at day 5 after infection. We observed substantial spreading of H3K9me2/3 and strong gene repression. Surprisingly, after loss of expression of the targeted methyltransferases, the induced epigenetic marks were lost over several days and the VEGF-A gene was reactivated to its original level. Interestingly, after targeted histone H3K9 methylation, we observed an excessive increase of histone acetylation during reestablishment of gene expression, suggesting that a response of the epigenetic network led to loss of the silencing marks and gene reactivation. Our data show that targeted chromatin repression of endogenous loci is attainable but difficult to maintain. It is likely that for preservation of the repressed state either multivalent deposition of functionally related epigenetic marks or longer-lasting trigger stimuli are necessary.

## Results

### Establishment of an adenoviral system for delivery of targeted DNA and H3K9 methyltransferases

**Figure 2:**
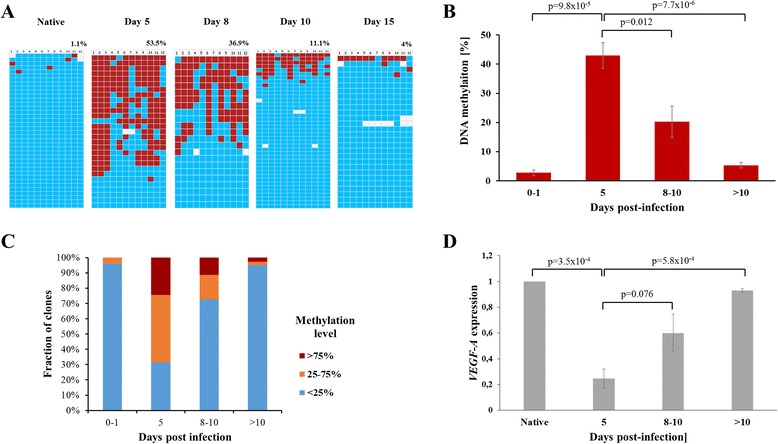
**DNA methylation-dependent induction of silencing. (A)** Example of the time-dependent deposition and loss of DNA methylation analyzed by bisulfite sequencing. Each row indicates one sequenced clone and each column indicates one CpG site. The blue and red colors display unmethylated and methylated CpGs, respectively. **(B)** Quantification of DNA methylation over time. The averages and SEM refer to the data sets shown in Additional file 1: Figure S2. **(C)** Methylation level ranking based on the data sets shown in Additional file 1: Figure S2. **(D)** Quantification of VEGF-A expression normalized to SDHA. The averages and SEM are based on two to three biological repeats. VEGF-A, vascular endothelial growth factor A.

Based on the novel achievements in genome targeting, including the discovery of the CRISPR-Cas9-targeting system, epigenetic reprogramming has moved into the center of research [18,28,29]. However, up to now, the stability of targeted epigenetic rewriting has not been studied in depth. We used chimeric DNA and H3K9 methyltransferases consisting of a zinc finger (ZF) domain that recognizes a 9-bp motif in the VEGF-A promoter coupled to the catalytic domains of the DNA methyltransferase 3a (Dnmt3a) (ZF-Dnmt3a-CD) or the ZF-protein lysine methyltransferase 1D (GLP) H3K9 methyltransferase (ZF-GLP-CD) for targeted epigenetic rewriting of the VEGF-A locus (Figure 1A,B,C). Our aim was to study the kinetics and stability of targeted DNA and H3K9 methylation after transient expression and loss of these targeted methyltransferases. Moreover, we studied the chromatin response to the targeted methylation and changes in the VEGF-A gene expression. The VEGF-A gene encodes for a signal protein involved in angiogenesis that has a critical role in cancer growth and metastasis [30]. In SKOV3 cells, the VEGF-A gene is expressed and its promoter is not methylated (Figure 2). The chimeric methyltransferases were delivered into the cells by adenoviral vectors. These vectors do not express the adenoviral E1 and E3 proteins, which are essential for the generation of viral particles [31]. Hence, they can infect SKOV3 cells but cannot propagate in these cells and are degraded after some time. The constructs also express green fluorescence protein (GFP) as a marker for tracking of infection and expression of the virus-encoded genes. Upon adenoviral delivery to SKOV3 cells, we generally observed infection of >95% of all cells based on GFP fluorescence (Additional file 1: Figure S1B). Fluorescence-activated cell sorting (FACS) analyses of GFP fluorescenceshowed that the expression of the virus-encoded genes increased after infection until a maximum was reached at day 5. Afterwards, the expression of virus-encoded genes declined, and it was almost lost after 10 days (Figure 1D).

### Silencing induced by targeted DNA methylation is not maintained

In a previous study, we have shown that transient transfection of a plasmid encoding a DNA methyltransferase fused to the VEGF-A-binding zinc finger into SKOV3 cells led to an establishment of DNA methylation and gene silencing [20]. However, with transient transfection, even after MACS purification of transfected cells, the maximal transfection rates were not higher than 50% to 80%. We wanted to extend this study in two ways: first, our aim was to introduce the chimeric DNA methyltransferase (ZF-Dnmt3a-CD) into the vast majority of cells via adenoviral delivery. Second, following the adenoviral infection, we wanted to monitor the changes in DNA methylation and expression state of the VEGF-A promoter over time. After ZF-Dnmt3a-CD delivery, we observed a gradual elevation of DNA methylation from day 1 after infection onwards (Additional file 1: Figure S2 and Figure 2A,B,C). The methylation signal peaked at day 5 with similar methylation levels, as observed previously after transient transfection of a similar targeting construct [20]. Since H3K9me3 is a silencing mark correlated with DNA methylation, we investigated if DNA methylation would induce deposition of H3K9 methylation. However, chromatin immunoprecipitation quantitative PCR (ChIP-qPCR) analyses of mononucleosomes isolated at day 5 from the infected cells showed no changes of H3K9me3 methylation, indicating that a crosstalk did not occur (Additional file 1: Figure S8A). After the 5-day period of incremental increase in DNA methylation, we observed a gradual loss of DNA methylation, which returned to its basal levels at day 15 (Additional file 1: Figure S2 and Figure 2A,B,C), indicating that DNA methylation levels directly correspond to the expression levels of the virus-encoded targeted DNA methyltransferase (Figure 1D). We also monitored the levels of VEGF-A gene expression upon deposition of DNA methylation. Interestingly, we observed a strong reduction of mRNA levels, most notably at day 5, followed by a gradual re-activation of the gene until day 10 to 15 (Figure 2D). Hence, the time course of VEGF-A gene downregulation and re-expression mirrored exactly the changes of DNA methylation in the VEGF-A promoter (Figure 2). The specificity of the targeted DNA methylation and repression was confirmed with several control viruses, including one construct in which the zinc finger was fused to a catalytically inactive Dnmt3a-CD variant (ZF-Dnmt3a-CD E752A) and constructs with ZF only, untargeted Dnmt3a-CD or empty vector (EV) (Additional file 1: Figure S3). To conclude, these data indicate a direct link between deposition of DNA methylation and gene silencing, with methylation and silencing levels comparable to previous results [20]. However, somehow unexpectedly, both the methylation and gene repression were not maintained upon loss of the targeted fusion DNA methyltransferase.

### Silencing can be induced by targeted H3K9 methylation but it is not maintained as well

**Figure 3:**
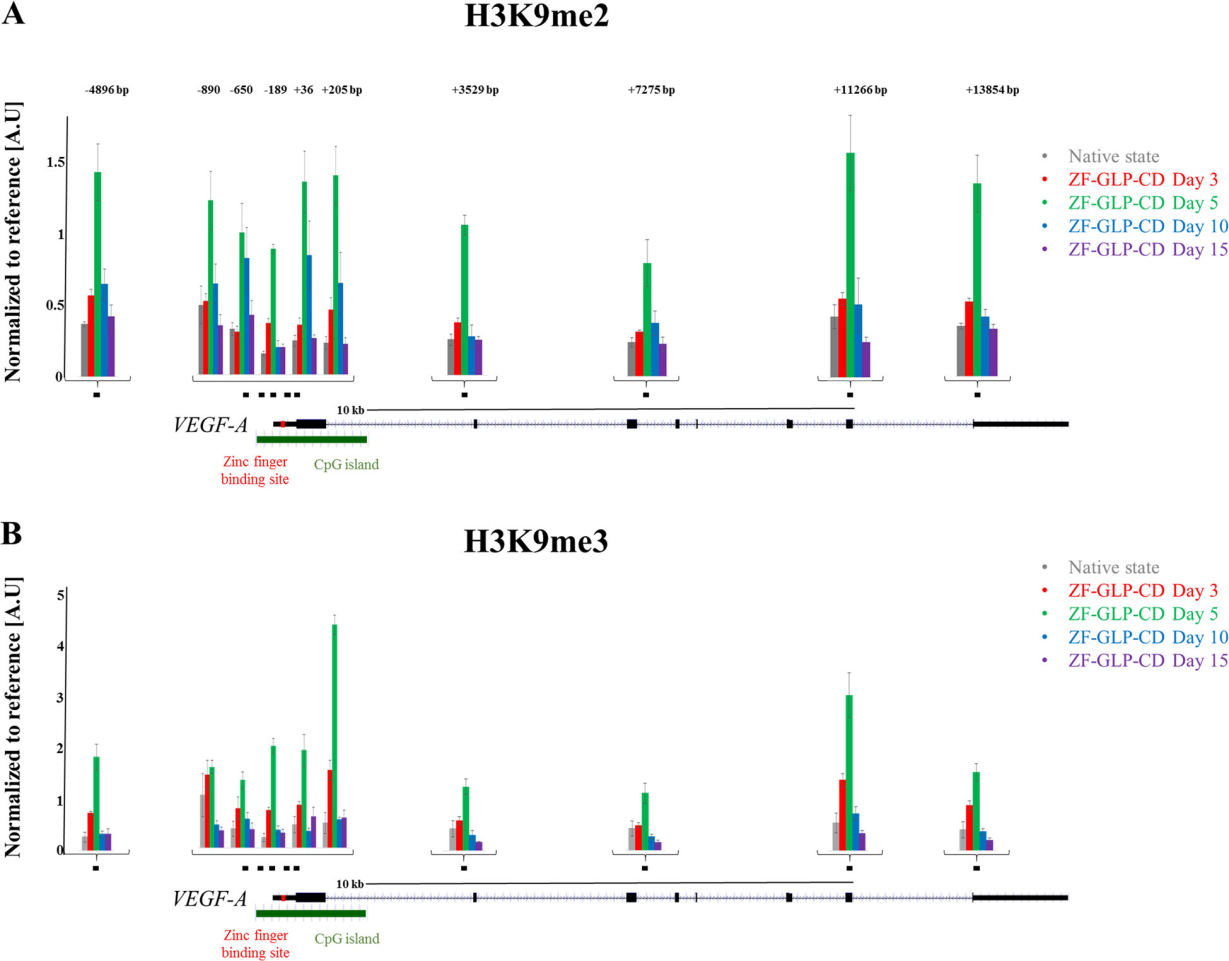
**Targeted deposition, spreading, and loss of histone H3K9 methylation over time.** The black boxes indicate the location of the amplicons used for ChIP-qPCR (also compare with Figure 1B for a larger image of the VEGF-A gene promoter). The numbers above the bars indicate the distance of the qPCR amplicons from the zinc finger-binding site. **(A)** ChIP-qPCR measurements of H3K9me2 at the designated amplicons covering the promoter and gene body of VEGF-A. **(B)** ChIP-qPCR measurements of H3K9me3 at the designated amplicons covering the promoter and gene body of VEGF-A. The averages and SEM are based on two to three biological repeats. The corresponding *P* values can be found in Additional file 1: Table S2. VEGF-A, vascular endothelial growth factor A; bp, base pair; ZF-GLP-CD, zinc finger-protein lysine methyltransferase 1D-catalytic domain.

**Figure 4:**
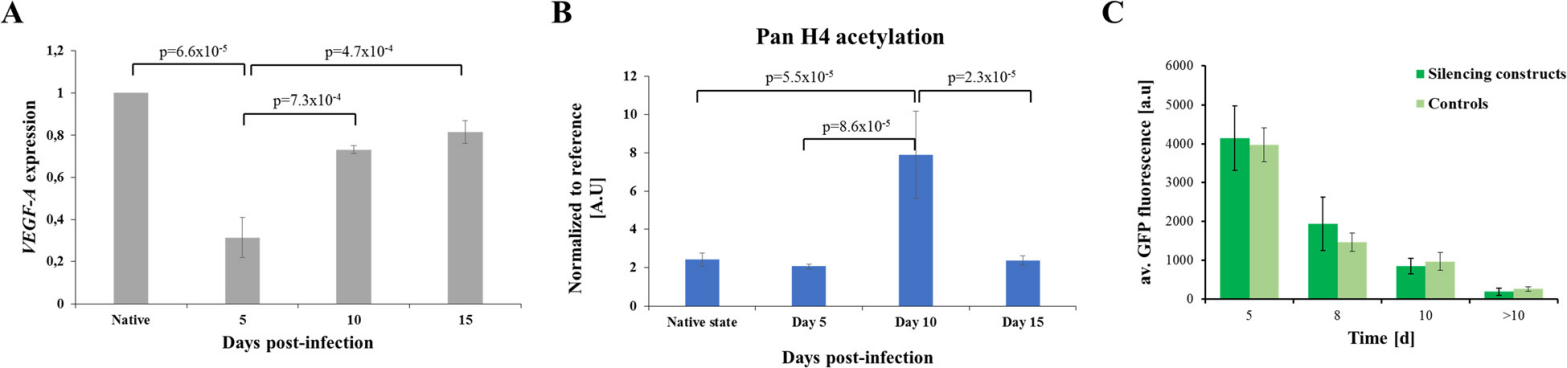
**H3K9 methylation-dependent induction of silencing. (A)** Quantification of VEGF-A expression normalized to SDHA. The averages and SEM are based on two to three biological repeats. **(B)** ChIP-qPCR measurements of H4 acetylation at the promoter of VEGF-A. The averages and SEM are based on two to three biological repeats. **(C)** Time course of GFP of all experiments analyzed in this respect. The dark green bars show the average data of experiments which led to VEGF-A silencing (*N* = 7). The light green bars show the averages of the controls not leading to silencing (*N* = 14). The error bars indicate the standard errors of the mean. VEGF-A, vascular endothelial growth factor A; GFP, green fluorescence protein; d, days.

The question whether histone PTMs are drivers or consequences of chromatin processes is a cardinal scientific issue that has been hotly debated [32]. For this reason, we wanted to directly study the effect of the targeted installment of an individual histone PTM on gene expression and to investigate the stability of this modification after removal of the epigenetic modifier. To this aim, we fused the catalytic domain of the GLP H3K9 methyltransferase with the zinc finger protein binding to the VEGF-A promoter (ZF-GLP-CD) and cloned this into an adenoviral vector. After adenoviral delivery of the targeted H3K9 methyltransferase, we followed the time course of H3K9 methylation and VEGF-A gene expression. We observed a gradual deposition of H3K9me2 and H3K9me3 at the target site (Figure 3). Interestingly, we also detected a massive spreading of both marks at least 5 kb upstream and 15 kb downstream from the zinc finger-binding site (Figure 3). Like DNA methylation, H3K9 methylation peaked at day 5, which was followed by return to its basal levels, indicating that the mark was not stably maintained. While H3K9me3 returned to initial levels at day 10, the kinetics of disappearance were slower for H3K9me2, which returned to its native state only at day 15 (Figure 3). This finding suggests a stepwise histone demethylation after the loss of expression of the targeted methyltransferase, in which H3K9me2 is generated as an intermediate by demethylation of H3K9me3. We also analyzed the gene expression of VEGF-A after targeted H3K9 methylation and found strong gene silencing at day 5 (Figure 4A). However, the trend of gene repression mirrored the deposition of H3K9me3 trimethylation and expression was reestablished fully at day 15 (Figure 4A). In addition, we measured the levels of histone H4 acetylation 200 bps downstream from the zinc finger-binding site and observed a strong surge of histone H4 acetylation at day 10 when the expression of the targeted methyltransferase had declined (Figure 4B,C). This effect was not due to changes in nucleosome occupancy, as determined by MNase mapping (Additional file 1: Figure S5). This result suggests that a histone acetyltransferase activity is recruited to the VEGF-A promoter by undetermined mechanisms. Similarly as before, we did not observe positive feedback between DNA methylation and H3K9 methylation, since no changes in DNA methylation levels were observed after targeted H3K9 methylation (Additional file 1: Figure S8B).

The specificity of our targeted epigenome editing was confirmed in several control experiments with zinc finger only or untargeted GLP catalytic domain, which both showed only residual silencing (Additional file 1: Figure S4). In an additional set of control studies, the zinc finger protein was fused with two different catalytically inactive GLP variants (ZF-GLP-CD C1201A and ZF-GLP-CD ΔNHHC) (Additional file 1: Figure S4). These constructs differ in their ability to interact with the endogenous G9a H3K9 methyltransferase which forms a heterodimeric complex with GLP in cells [33]. The GLP C1201A variant is still able to form heterodimers with G9A but the GLP-CD ΔNHHC is not. The slightly stronger residual silencing of VEGF-A expression observed with the GLP C1201A variant suggests that a recruitment of native G9A by the chimeric ZF-GLP-CD protein has some enhancing effect on gene repression in our system. We also detected a slight increase of H3K9me3 at the VEGF-A locus with untargeted GLP-CD (without zinc finger) at day 3, which declined to basal levels at day 5 unlike ZF-GLP-CD, which peaked at day 5 (Additional file 1: Figure S6-S7 and Figure 3). To conclude, our data indicate that the downregulation of VEGF-A is a direct consequence of H3K9me2/3 deposition and spreading and suggest that these particular histone marks likely have a direct role in the regulation of gene expression. In addition, as in the case of DNA methylation, the H3K9 methylation mark was not stably maintained and the observed gene silencing was only transient. Interestingly, our data also suggest that the surge of histone acetylation at day 10 is likely a feedback ‘over-reaction’ from the cellular epigenetic network, which overrides the repressive state and leads to gene reactivation.

### Loss of DNA and H3K9 methylation is not due to a growth disadvantage of cells caused by VEGF-A silencing

We wanted to determine if the loss of the DNA and H3K9 methylation signal after 5 days could be caused by a selective growth disadvantage of cells after VEGF-A silencing. However, the gradual loss of DNA and H3K9 methylation and gene silencing observed after day 5 was not correlated with a shift of cell populations. This is illustrated by the observation that the time course of loss of GFP expression was identical in cells infected with the various control viruses (such as EV, virus encoding ZF alone, or viruses that encode for catalytically inactive methyltransferases) and viruses that lead to DNA or H3K9 methylation and corresponding gene silencing (ZF-Dnmt3a-CD or GLP-CD virus) (Figure 4C). These data indicate that the relative proportions of cells with or without the adenoviral vector did not change regardless of whether the VEGF-A gene was silenced or not. Hence, the loss of the DNA and H3K9 methylation mark reflects a true loss of the signal and not a diminishment of the cell population that carries the signal.

## Discussion

The epigenome of each cell from a multicellular organism is a highly dynamic entity that realizes the transcriptional programs encoded within the genome, balancing between phenotypical stability and environmental plasticity. In this study, we were able to provide a valuable insight into the inner workings of chromatin by setting a defined chromatin modification at an endogenous locus and following the dynamics of its appearance and disappearance with a spatial and temporal resolution. Previous studies have successfully designed and applied modular DNA-binding proteins fused with eukaryotic or prokaryotic DNA methyltransferase activities for targeted DNA methylation [34]. Notably, the technology involving mammalian Dnmt catalytic domains fused to zinc finger proteins was successfully used for targeted methylation of endogenous promoters of cancer-associated genes, including VEGF-A [20-22]. In previous studies performed in our lab, transient transfection methods were employed for delivery of the targeted DNA methyltransferases. This allowed to transfect about 50% to 80% of all cells leading to significant DNA methylation and gene downregulation of the tested genes (EpCAM or VEGF-A) [20,22]. In the current work, we extended these experiments and used an adenoviral delivery system, which has significantly improved the number of infected cells expressing the targeted DNA and H3K9 methyltransferases. We observed a clear anti-correlation between gene expression and the DNA and H3K9 methylation levels, suggesting that these epigenetic marks have a direct effect on gene regulation. The viral delivery system enabled us to follow the dynamics of the establishment and loss of the H3K9 and DNA methylation in the VEGF-A locus, and interestingly, we observed a lack of preservation of both marks after loss of expression of the respective targeted methyltransferase. Our results indicate that activating signals from the surrounding chromatin domain of the VEGF-A gene overruled the local silencing signal and led to its disappearance.

Different from our results, in a previous report, DNA methylation introduced at the *Maspin* locus was stable over multiple generations [21]. One explanation for this discrepancy could be the chromatin context of the loci tested, with one being more permissive to permanent silencing than the other. This difference may also be attributed to the technical approach, because in the Rivenbark study, stable cell lines were generated, which contained the gene of the targeted methyltransferase. While it was found that the expression levels of the chimeric methyltransferase were drastically reduced after several cell generations, it is still conceivable that a small amount of the effector was still present in the cell, sufficient for the maintenance of the silenced state.

Only a few studies prior to this one showed promise in testing the utility of ZF-targeted histone methyltransferases in epigenome editing [26,27,35]. Apart from validating the general feasibility of this approach, we also monitored the dynamics of the establishment and disappearance of the H3K9 methylation and show lack of preservation of H3K9 methylation. Moreover, we show spreading of the modification for at least 15 kb downstream and 5 kb upstream from the nucleation site. Similar observations of heterochromatin spreading were made by chemically induced recruitment of the HP1 chromo shadow domain to an artificial promoter [36]. In this study, the heterochromatization was inherited only after a prolonged chemical stimulus (4.5 weeks), which led to a local gain of DNA methylation. Similar to our study, the presence of chemical stimulus for 3 to 7 days, only led to a transient deposition of H3K9me3 and reduced transcriptional output, both of which were lost after cessation of the inducing stimulus. In another study in *Schizosaccharomyces pombe* strains, a heterochromatic block of H3K9 methylation was successfully established but lost over days which is in agreement with our findings [35].

## Conclusions

**Figure 5:**
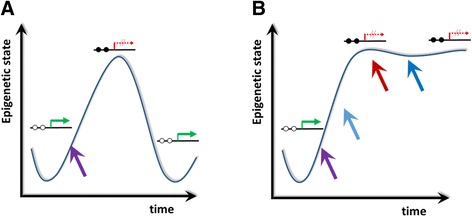
**Conclusions for the reprogramming of epigenetic states. (A)** Individual stimuli (indicated by the purple arrow) can edit the epigenetic state and silence gene expression but the silenced state is not stable. **(B)** For stable epigenetic silencing, presumably multiple triggers or individual triggers lasting over longer times are needed.

The data obtained in our experimental system indicate that the epigenetic chromatin state can be easily edited by setting a trigger ‘repressive’ mark, concurrently leading to gene silencing. Interestingly, after discontinuance of the trigger signal (both in the case of DNA and H3K9 methylation), the chromatin state and transcriptional levels shifted back to their native state. This suggests that the maintenance and mitotic inheritance of an epigenetic mark, even DNA methylation, which is considered to be one of the most stable silencing signals, might not be a trivial task. This finding is in agreement with recent observations suggesting that the initial model of DNA methylation inheritance in a maintenance process was an oversimplification, and that DNA methylation is better described by a dynamic and stochastic model [37]. Our data suggest that epigenetic states are encoded in a network of epigenetic marks, and they cannot be reset easily by rewriting a single mark. To achieve this, a multivalent deposition of functionally related epigenetic marks or longer-lasting trigger stimuli might be necessary (Figure 5). The epigenetic network provides feedback regulatory mechanisms; and processes such as imprinting, heterochromatization, or housekeeping gene transcription are most likely continuously supported with balancing regulators in order to preserve their initial chromatin state. Furthermore, our data show that DNA methylation and H3K9 methylation did not enforce each other and the targeted H3K9 methylation and gene silencing did not reduce H4 acetylation. It would be interesting to see if additional treatment of cells with demethylase inhibitors, generation of longer-lasting trigger signals, or combined deposition of DNA and histone methylation could induce mitotic stability and true epigenetic inheritance. In addition, it is an interesting question to study the effect, stability, and network response of the targeted deposition of an ‘activating’ chromatin mark, such as H3K4me3 or histone acetylation. Finally, with the development of novel genome targeting technologies, it will be compelling to observe the effects of setting or erasing single or combinatorial epigenetic marks in different chromatin contexts. For this, it will be necessary to study the stability of epigenetic reprogramming at several loci and learn about the influence of the chromatin environment on the stability of gene silencing. This will pave the way into true epigenetic editing in the sense that the newly generated epigenetic states would be as stable as the natural ones and have comparable biological effects.

## Methods

### Cloning and production of recombinant adenovirus

The zinc finger protein which binds a 9-bp sequence (GGGGGTGAC) in the VEGF-A promoter [26], ZF fused to Dnmt3a-CD (amino acids 608-908, UniProt no. O88508), or Dnmt3a-CD alone were amplified and sub-cloned into the pAdTrackCMV vector using the BglII and XhoI restriction sites. The DNA fragments containing the catalytic domain, H3K9 methyltransferase G9a-like protein (amino acids 1002-1295, UniProt no. Q96KQ7), were cloned in empty or zinc finger-containing pAdTrackCMV vector using the SalI and HindIII restriction sites. The production of adenoviral vectors and adenovirus was based on [31]. Briefly, the pAdTrackCMV vector containing the gene of interest was linearized with PmeI and was co-transformed with pAdEasy-1 vector into *Escherichia coli* BJ5183. Successful recombination was confirmed by restriction digestion and Sanger sequencing. Six micrograms of PacI linearized vector was transfected into HEK293 cells in T-25 flask. HEK293 are E1 positive, thus allowing virus production. After transfection, the HEK293 cells were maintained in DMEM supplemented with 5% fetal bovine serum in a CO_2_ incubator at 37°C for 14 to 21 days, with addition of 2 ml medium, every 4 days. The adenoviral vector also expressed GFP to allow for the FACS analysis of infection yields and follow the expression levels of virus-encoded genes. The expression of GFP and the targeted zinc finger-fused methyltransferases was both driven by a CMV promoter. Total infection was confirmed microscopically, and viral lysates were prepared for high-titer virus production. In the end, the mature virus was collected using cesium chloride (CsCl) density gradient centrifugation and gel filtration (Nap™ columns, GE Healthcare, Pewaukee, WI, USA), and it was used for further infections. The optimal viral titer for infection of SKOV3 cells was determined by serial dilutions.

### Infection of SKOV3 cells with recombinant adenoviral vectors

SKOV3 cells were obtained from ATCC (American Type Cell culture Collection) and cultured in DMEM supplement with 10% fetal bovine serum, l-glutamine, and penicillin/streptomycin. SKOV3 cells were seeded in a density of 2 × 10^5^ cells per well in a six-well plate, and the following day the cells were infected with the adenoviral vectors. Virus dilutions were selected to yield >95% of infection without affecting the cell viability. The infection yield was determined by measuring GFP fluorescence (FACSCalibur, BD Biosciences, San Jose, CA, USA), where uninfected cells were used as a control. One day post-infection, the free virus was removed and the cells were washed with warm PBS. The samples were collected by trypsinization at day 3 or day 5 post-infection. Half of the cells harvested at day 5 was propagated until day 10 or day 15. For chromatin isolation, the whole protocol was up-scaled to 2 × 10^7^ cells. Corresponding samples were always used for bisulfite sequencing, chromatin immunoprecipitation, and gene expression experiments. The generation of adenoviral particles and infection of SKOV3 was done in compliance with Biosafety Level 2 regulations.

### Analysis of methylation by bisulfite conversion

Genomic DNA was isolated using the QIAmp® DNA mini kit (Qiagen, Limburg, The Netherlands). Four hundred nanograms of genomic DNA were digested with BamHI overnight at 37°C, and bisulfite conversion was carried out using sodium bisulfite and sodium hydroxide as described [38]. After bisulfite conversion the genomic DNA was concentrated and purified using Amicon filters (Millipore, Billerica, MA, USA) and amplified using the following primers: FP 5′-GTT TGT TAT TTT TTA TTT GAA T-3′ and RP 5′-AAT CAC TCA CTT TAC CCC TAT C-3′ [20]. The PCR product was subcloned using the Strataclone PCR cloning kit (Agilent Technologies, Santa Clara, CA, USA) and several individual clones were sequenced.

### Native chromatin immunoprecipitation (nChIP) and gene expression

Native mononucleosomes were prepared from around 20 million SKOV3 cells by micrococcal nuclease digestion of nuclei as described [39] with minor modifications. More precisely, after MNase treatment, the nuclei were spun down at 13,000*g* for 10 min, and the soluble nucleosomal supernatant was collected and snap frozen. We used 10 to 15 μg (based on DNA absorbance) of pre-cleared native chromatin per ChIP with anti-H3K9me2 (ab1220, Abcam plc, Cambridge, UK), anti-H3K9me3 (ab8898, Abcam plc, Cambridge, UK), or pan-H4-acetyl (AM 39243) (go to Additional file 1: Figure S5 to see their peptide array specificity profiles). After immobilization on protein G-coated magnetic Dynabeads (Invitrogen, Waltham, MA, USA), the antibody-chromatin complexes were washed with: 1× low salt buffer (20 mM Tris-Cl, 150 mM NaCl, 1% Triton × -100, 0.1% SDS, and 2 mM ethylenediaminetetraacetic acid (EDTA)), 1× high salt buffer (20 mM Tris-Cl, 500 mM NaCl, 1% Triton × -100, 0.1% SDS, and 2 mM EDTA), 1× LiCl buffer (10 mM Tris-Cl, 250 mM LiCl, 1% NP-40, 1% DOC, and 1 mM EDTA), and 2× TE buffer. After each wash, the beads were rotated for 10 min at +4°C. The bound nucleosomes were eluted in elution buffer (50 mM Tris-Cl, 50 mM NaCl, 1 mM EDTA, 1% SDS), for 45 min at room temperature with rotation. DNA was recovered using ChIP DNA purification columns (Active Motif, Carlsbad, CA, USA).

The quantitative PCR assays were performed on a CFX96 Connect Real-Time detection system (Bio-Rad, Hercules, CA, USA) using SsoFast EvaGreen supermix (Bio-Rad, Hercules, CA, USA). In the nChIP experiments, a standard curve was generated to calculate percent of precipitated DNA and test the efficiency of each primer set covering the VEGF-A locus. The primer sequences can be found in Additional file 1: Table S1. To correct for technical quality between the different samples, each amplicon signal was normalized to an internal positive control which carries the corresponding mark and is not affected by the reprogramming (satellite alpha or gene desert-12 amplicons in the case of H3K9me2/3 or PABPC1 amplicon in the case of pan-H4ac). To this end, the percent of precipitated DNA was calculated for each amplicon and then divided by the percent of precipitated DNA obtained with the amplicons from the internal positive control. For nucleosomal mapping, mononucleosomal DNA was compared to a standard curve from genomic DNA and normalized for technical variability to an internal control (HOX11).

For gene expression analyses, total RNA was isolated for each time-point using the Purelink™ RNA mini kit (Ambion, Life Technologies, Carlsbad, CA, USA) and cDNA was prepared with oligo d(T)_18_ primers (New England Biolabs, Ipswich, MA, USA) from 1 to 2 μg of RNA. After this, qPCR was carried out using VEGF-A specific primers (FP 5′-AGA AGG AGG AGG GCA GAA TCA-3′ and RP 5′-ATG GCT TGA AGA TGT ACT CG-3′), normalized to the housekeeping gene SDHA (FP 5′-TGG GAA CAA GAG GGC ATC TG-3′ and RP 5′-CCA CCA CTG CAT CAA ATT CAT-3′). Non-RT controls were included in all experiments, and the total VEGF-A expression was quantified using the 2^−ΔΔCT^ method (threshold cycle (C_T_)) [40].

### Data analysis

Data are reported as means of biologically independent experiments as indicated. Error bars indicate the corresponding standard error of the mean. *P* values were determined by Excel using two tailed *T* tests with equal variance.

## Additional file

Additional file 1:
**Supplementary Figures S1-S9 and Supplementary Tables S1 and S2.**

## Abbreviations

CD: catalytic domain
ChIP: chromatin immunoprecipitation
Dnmt3a: DNA methyltransferase 3a
EDTA: ethylenediaminetetraacetic acid
EpCAM: epithelial cell adhesion molecule
GFP: green fluorescence protein
GLP: protein lysine methyltransferase 1D
PTM: post translational modification
TALE: transcription activator-like effector
VEGF-A: vascular endothelial growth factor A
ZF: zinc finger
