# Supplementary material for: Targeted epigenome editing of an endogenous locus with chromatin modifiers is not stably maintained
Source: Epigenetics Chromatin. 2015 Mar 18;8:12. doi: 10.1186/s13072-015-0002-z (PMC4404288; doi:10.1186/s13072-015-0002-z)
Supplement: Additional file 1: — Supplementary Figures S1-S9 and Supplementary Tables S1 and S2. [file 13072_2015_2_MOESM1_ESM.pdf]

Targeted epigenome editing of an endogenous locus with chromatin modifiers is not stably maintained

Goran Kungulovski, Suneetha Nunna, Maria Thomas, Ulrich M. Zanger, Richard Reinhardt and Albert Jeltsch

Additional file 1 containing:

SUPPLEMENTARY FIGURES S1-S9

SUPPLEMENTARY TABLES 1 and 2

Figure S1. A) Snapshot of the *VEGF-A* promoter. The amplicon used for bisulfite DNA methylation analysis is shown in green, the ZF binding site in red and the qPCR amplicons in the promoter are shown in black. Also compare Fig. 2B. Potential transcription factors binding sites surrounding the ZF binding site within 500 bps were identified in the JASPAR database (http://jaspar.binf.ku.dk/) and sites with relative profile scores above 95% are indicated in grey. B) Representative examples of FACS analyses of infected and uninfected SKOV3 cells determined 5 days after adenoviral infection. GFP fluorescence showed consistently >95% fraction of infected cells.

A

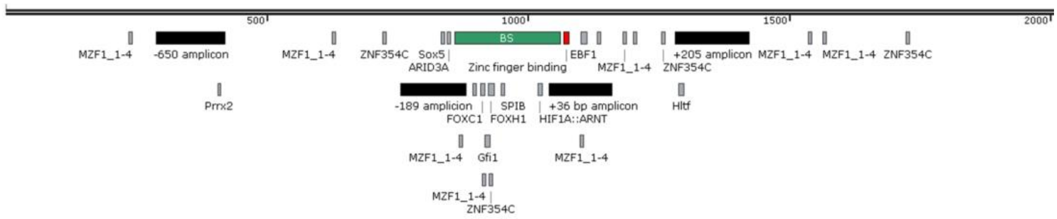

B

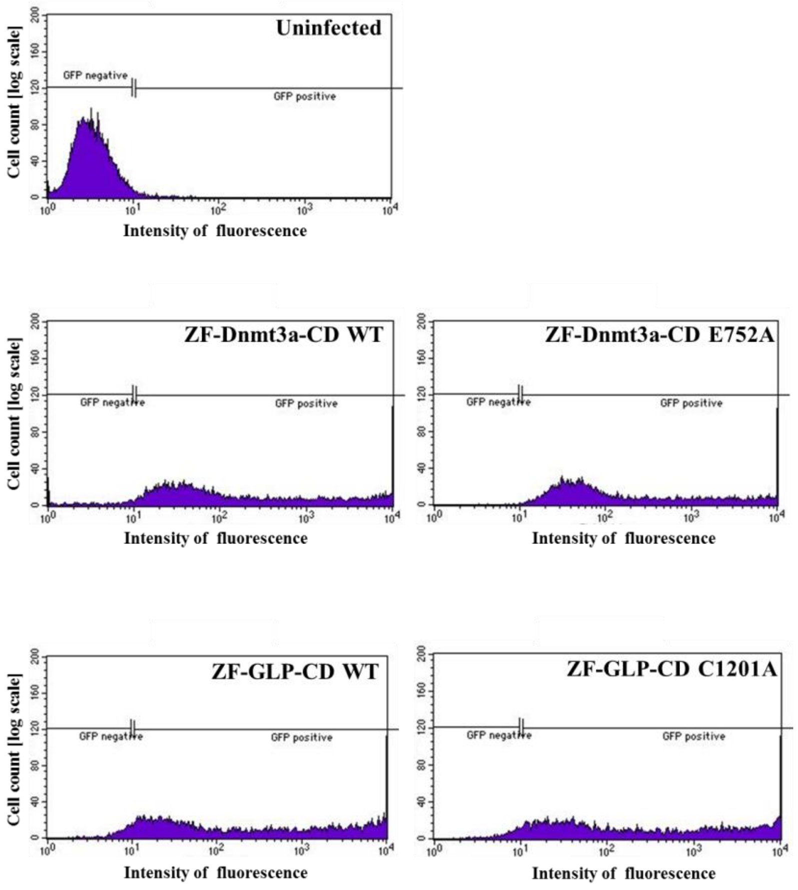

Figure S2. A) Time dependent deposition and loss of DNA methylation analyzed by bisulfite sequencing in five experiments. Each row indicates one sequenced clone, and each column indicates one CpG site. The blue and red color display unmethylated and methylated CpGs, respectively. B) Graphical representation of the data shown in A. Compilations of these data are shown in Figure 2B and C.

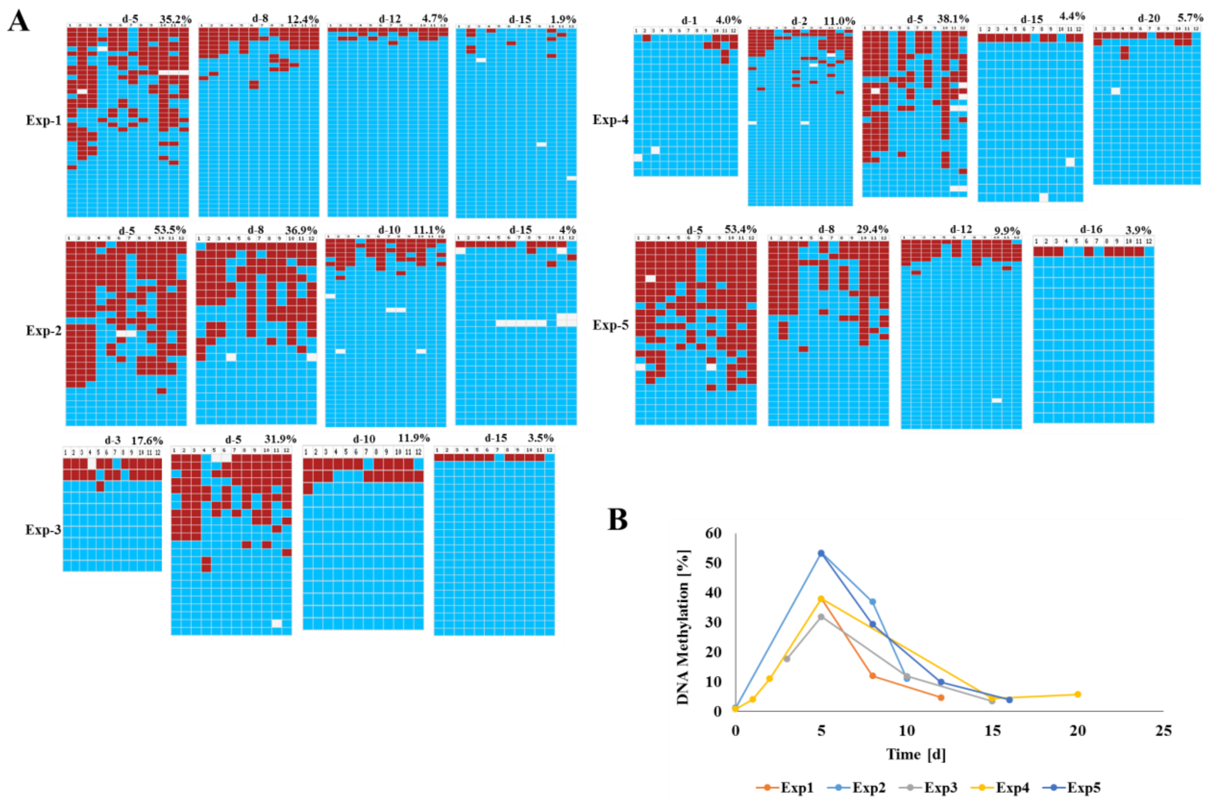

Figure S3. Control experiments demonstrating the specificity of the targeted DNA methylation. A) Similar GFP fluorescence at day 5 between the ZF-Dnmt3a-CD WT and E752A inactive mutant. B) Quantification of *SDHA* normalized *VEGF-A* expression of ZF, ZF-Dnmt3a-CD wild type and inactive mutant at day 5. The averages and SEM are based on 2-3 biological repeats. C) Lack of DNA methylation of different controls indicates the specificity of DNA methylation deposition.

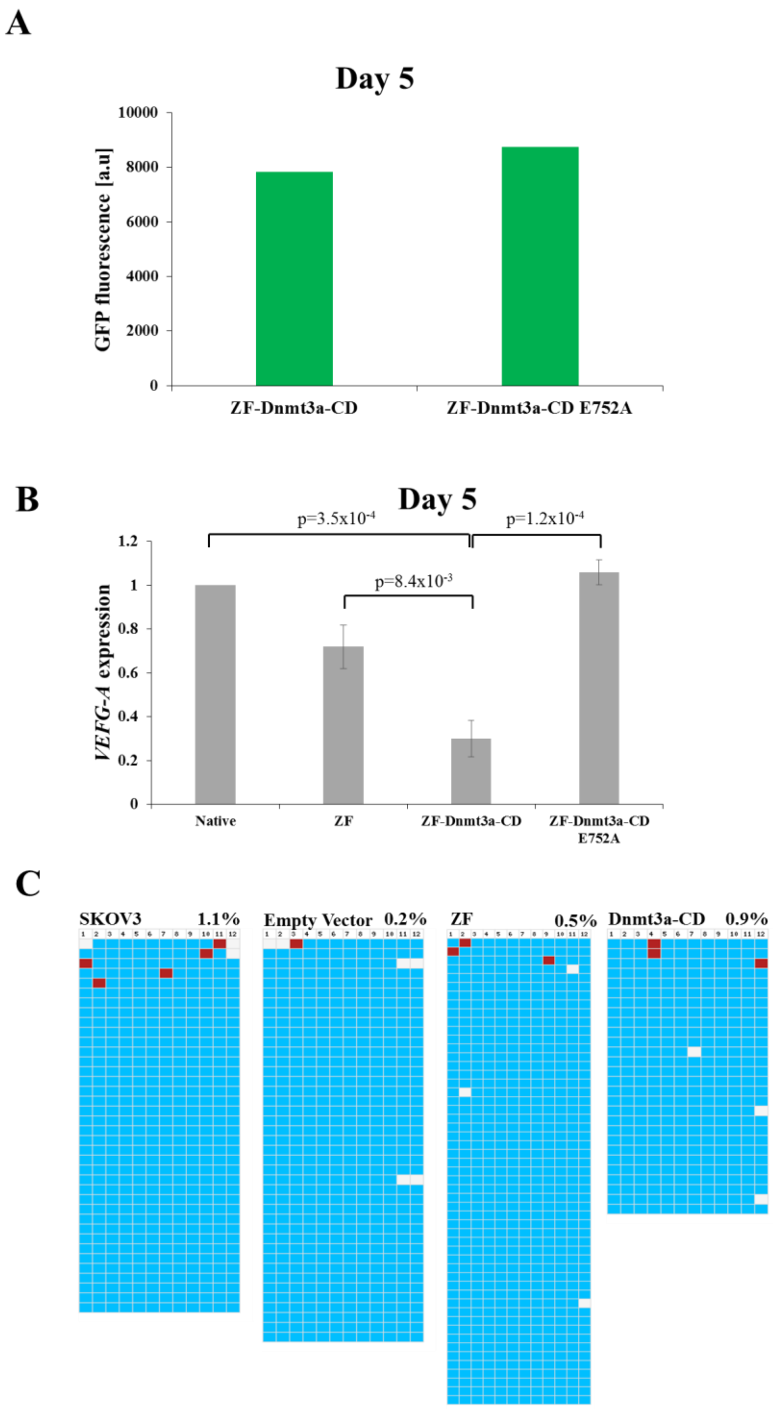

Figure S4. Control experiments demonstrating the specificity of the targeted histone H3K9 methylation. A) Similar GFP fluorescence at day 5 between the ZF-GLP-CD and ZF-GLP-CD inactive mutants. B) Quantification of *SDHA* normalized *VEGF-A* expression of ZF, ZF-GLP-CD wild type and inactive mutants at day 5. The averages and SEM are based on 2-3 biological repeats.

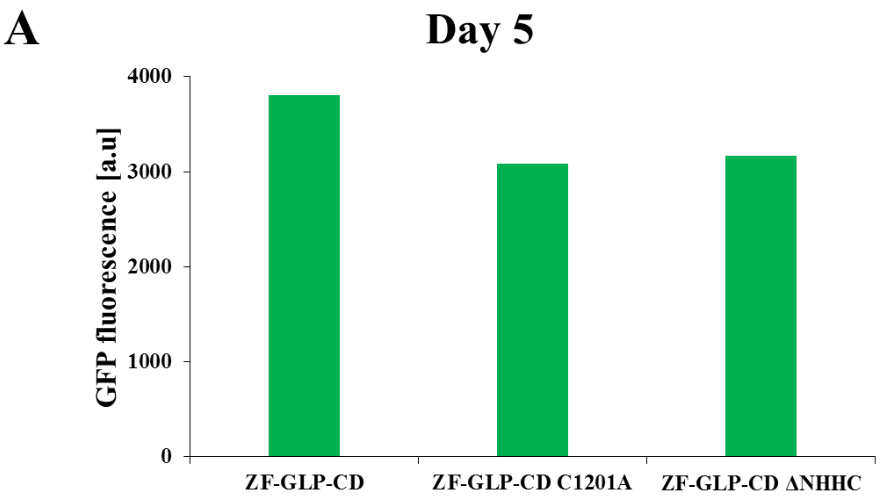

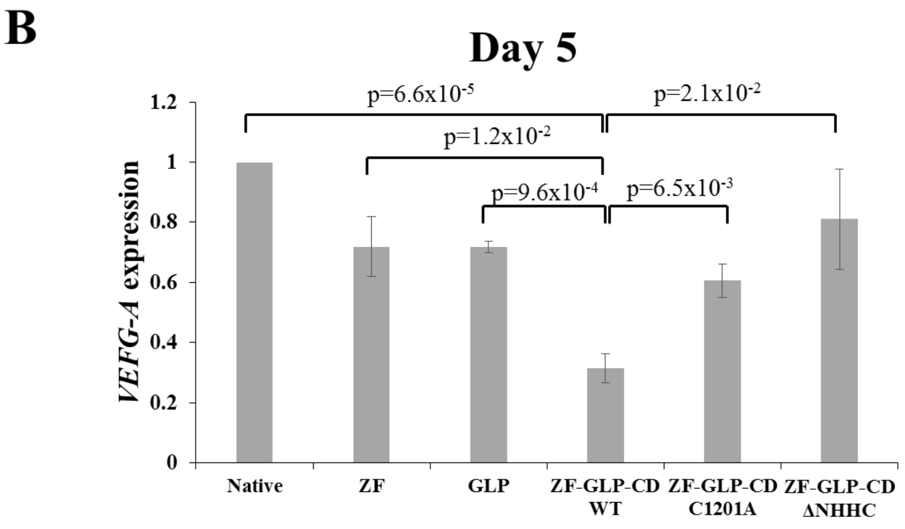

Figure S5. Nucleosome mapping quantified by MNase-qPCR shows no notable changes in nucleosomal occupancy. The averages and SEM are based on 2 biological repeats.

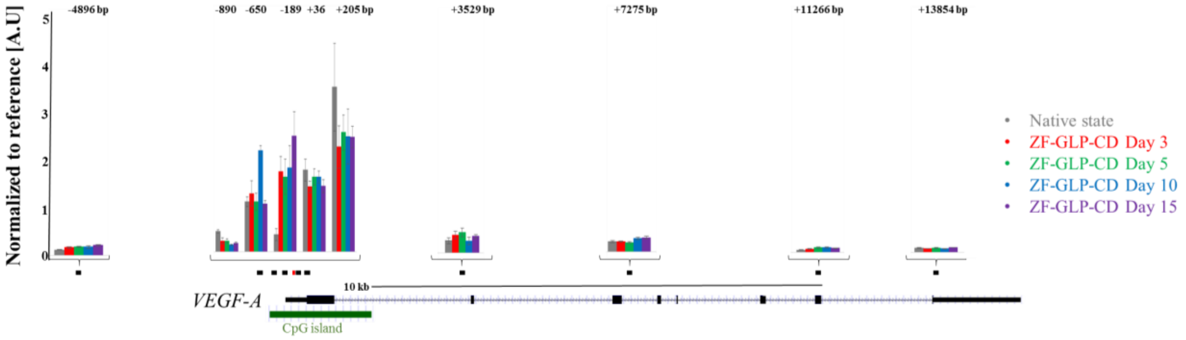

Figure S6. Time course of targeted deposition, spreading and loss of histone H3K9me3 methylation in control experiments. The red box denotes the zinc finger binding site, whilst the black boxes denote the amplicons used for ChIP-qPCR. The numbers above the bars show the distance of the qPCR amplicons upstream and downstream from the zinc finger binding site. A) ChIP-qPCR measurements of the *VEGF-A* locus for H3K9me3 presence in cells infected with ZF-GLP-CD. B) ChIP-qPCR measurements of the *VEGF-A* locus for H3K9me3 presence in cells infected with GLP-CD-only. C) ChIP-qPCR measurements of the *VEGF-A* locus for H3K9me3 presence in cells infected with ZF-only. The averages and SEM are based on 2-3 biological repeats.

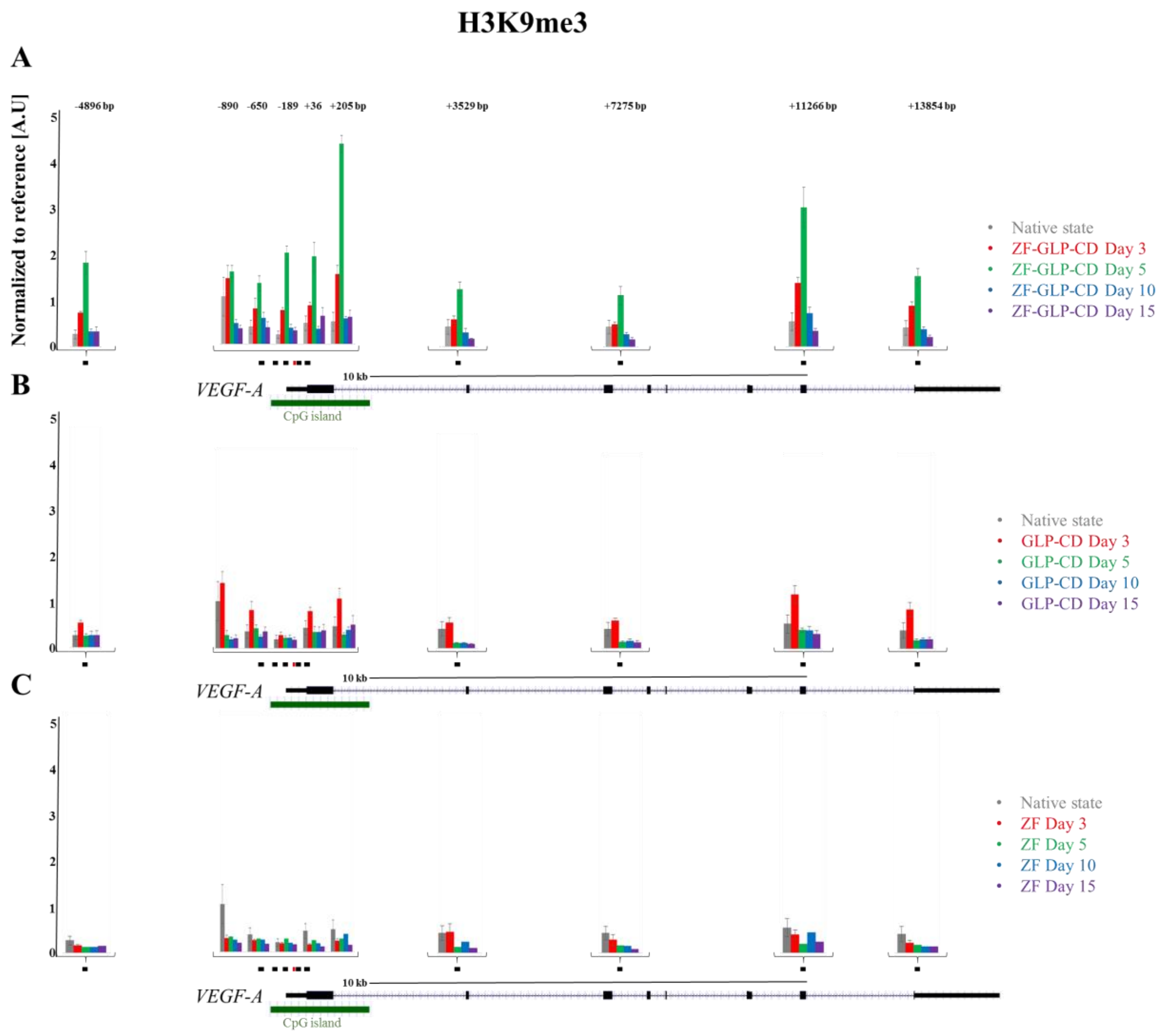

Figure S7. Time course of targeted deposition, spreading and loss of histone H3K9me2 in control experiments. The red box denotes the zinc finger binding site, whilst the black boxes denote the amplicons used for ChIP-qPCR. The numbers above the bars show the distance of the qPCR amplicons upstream and downstream from the zinc finger binding site. A) ChIP-qPCR measurements of the *VEGF-A* locus for H3K9me2 presence in cells infected with ZF-GLP-CD. B) ChIP-qPCR measurements of the *VEGF-A* locus for H3K9me2 presence in cells infected with GLP-CD-only. The averages and SEM based on 2-3 biological repeats.

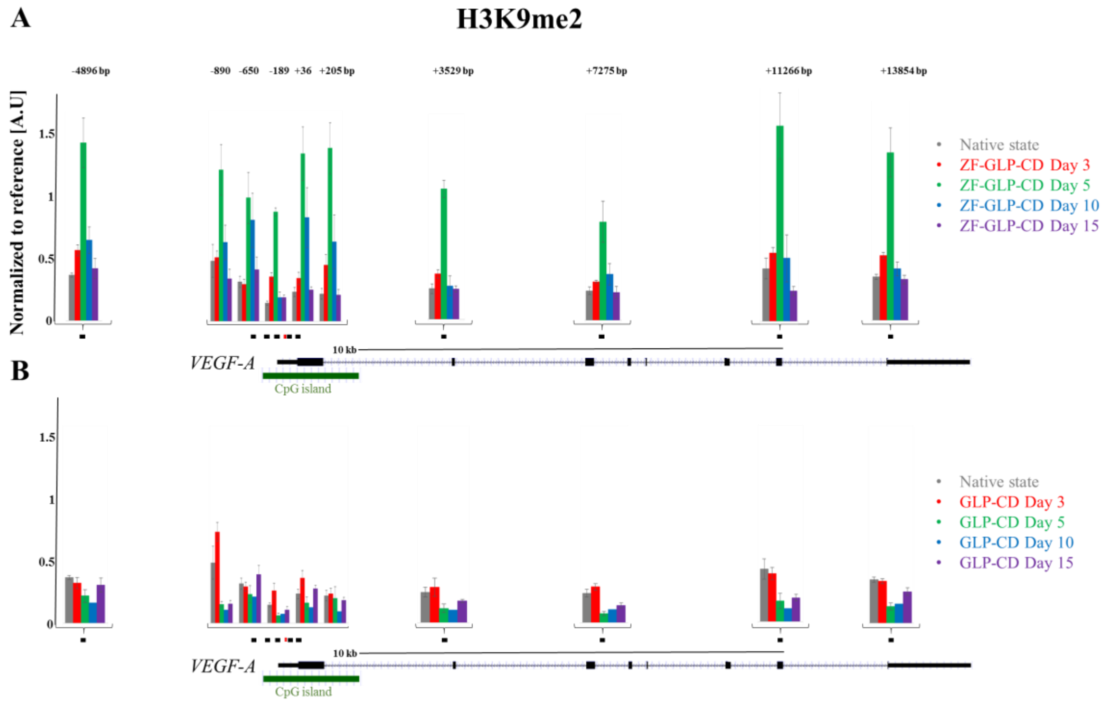

Figure S8. Absence of detectable cross-talk between DNA and H3K9 methylation. A) Targeted deposition of DNA methylation does not lead to subsequent histone H3K9me3. B) Additional bisulfite sequencing data showing absence of DNA methylation under conditions of dense H3K9me2/3 methylation (after transfection with ZF-GLP-CD). These results indicate that targeted deposition of histone H3K9 methylation does not lead to subsequent DNA methylation.

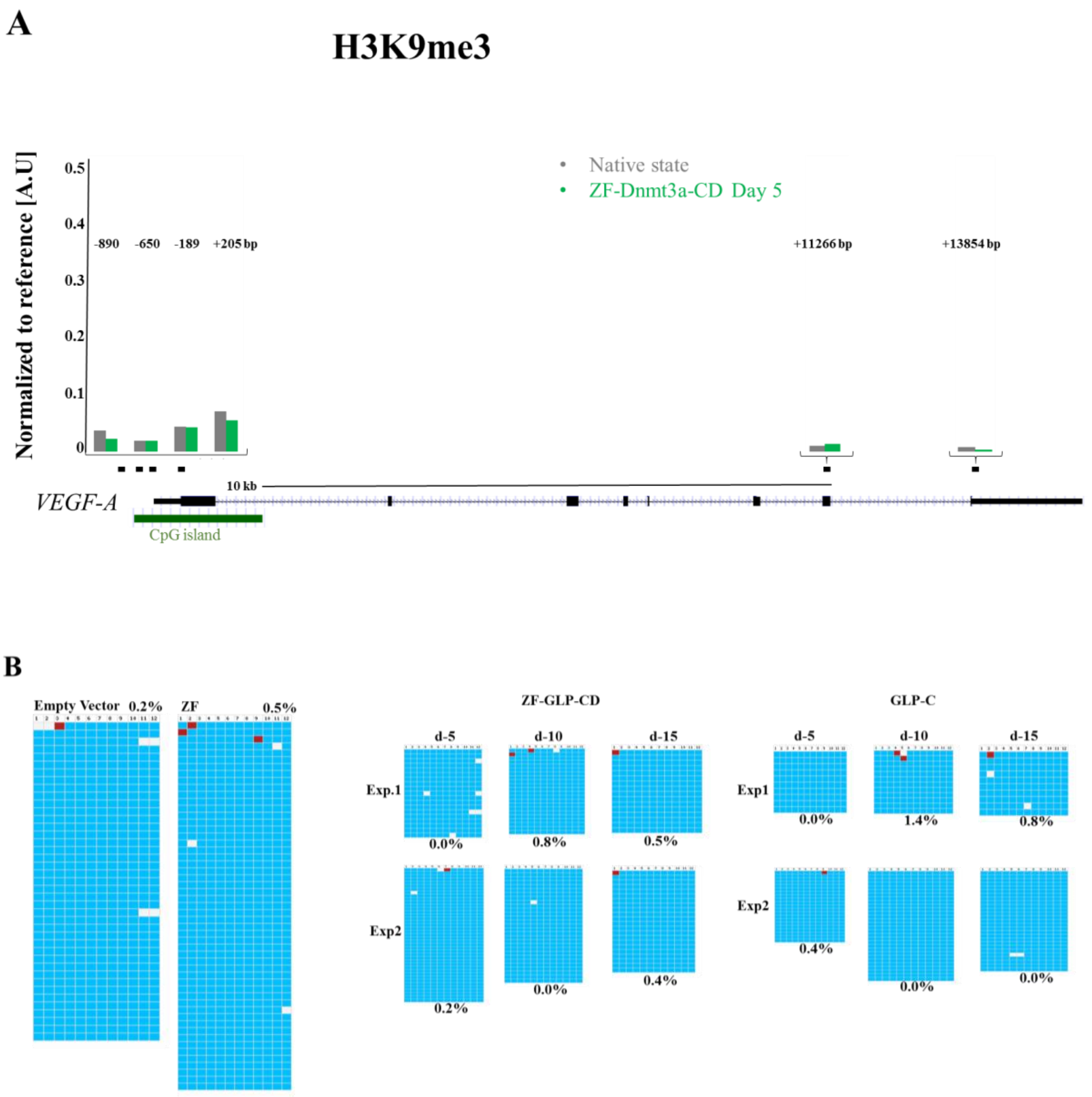

Figure S9. Peptide array specificity analyses of the antibodies used in this study. The peptide spots are annotated on the left side of the glass slide and the color coded boxes show the presence of the designated modified peptides. A) The first antibody (upper panel) bound primarily to H3K9me3 harboring peptides, with minor cross-specificity to H3K27me3 and H4K20me3. The second antibody (lower panel) bound to the targeted H3K9me3, but also showed notable cross-specificity with H4K20me3 peptides and minor cross-specificity with H3K27me3 peptides. B) The H3K9me2 antibody bound primarily to H3K9me2 peptides, with some cross-reactivity to H3K9me1 peptides. C) The anti-H4-pan-Ac antibody exhibited binding to acetylated H4 peptides, with minor cross-reactivity to H2A acetylated peptides.

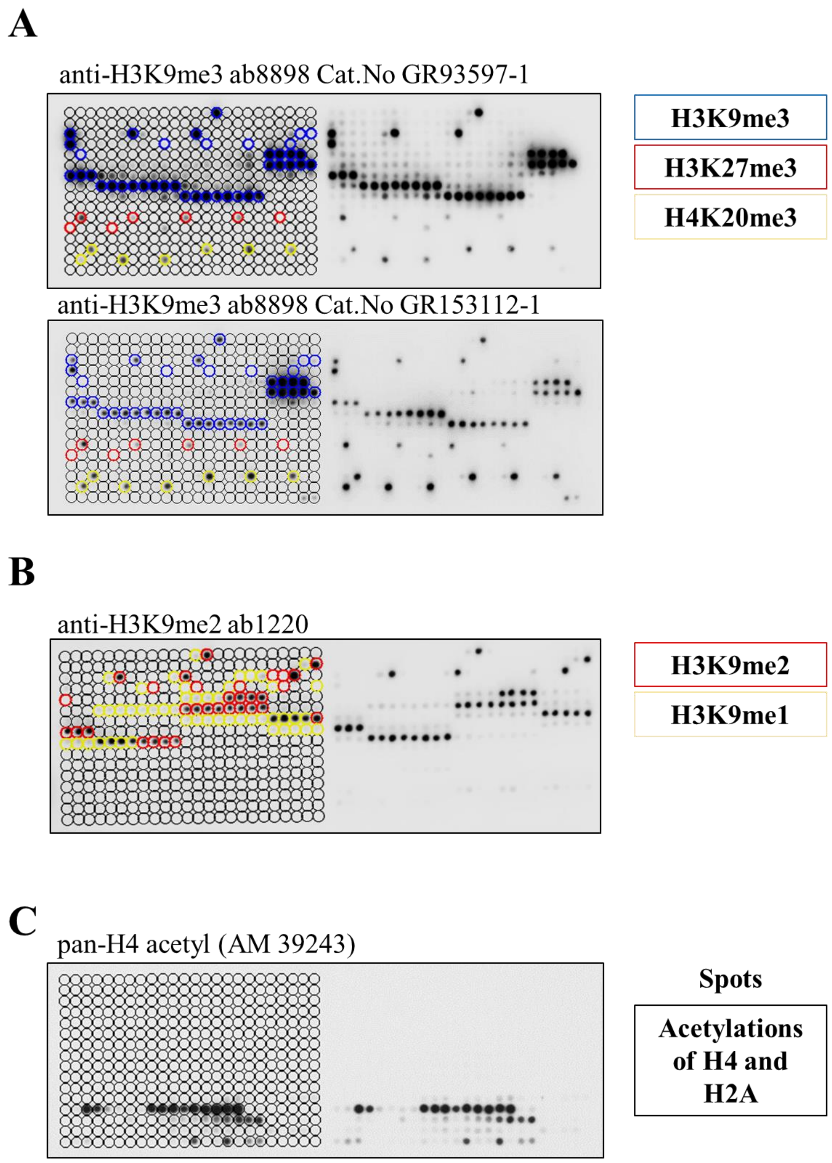

Table 1. List of primers used in nChIP-qPCR covering the *VEGF-A* locus.

Name	Primer sequences
-4896 bp	FP 5-GTAGTCCCAGGGTGCAACAC-3<br>RP 5-GACTGGCTAGAATGGGCATC-3
-890 bp	FP 5-GCAGTCACTAGGGGGCGCTC-3<br>RP 5-CAACGCCCTCAACCCCAC A-3
-650 bp	FP 5-CCTAGCAAAGAGGGAACGGCT-3<br>RP 5-AGCCTGAAAATTACCCATCCGC-3
-189 bp	FP 5-CGGTGCTGGAATTTGATATTCATTGAT-3<br>RP 5-TTCAAGTGGGGAATGGCAAGC-3
+36 bp	FP 5-ACAGGGGCAAAGTGAGTGAC-3<br>RP 5-GCGGTGTCTGTCTGTCTGTC-3
+205 bp	FP 5-CGCACTGAAACTTTTCGTCCA-3<br>RP 5-CTCCTCCCCCTCCTCCGG-3
+3529	FP 5-GAAGCATTCTGGGGTTTTCC-3<br>RP 5-GCACAGAATATGCCCACCAC-3
+7275	FP 5-GGTCTTGCCTTCTGTGGAGA-3<br>RP 5-ACACAGGCATCCATCTGTCC-3
+11266	FP 5-GGGGTGTTGCATGGTGATTTTTT-3<br>RP 5-TGCGGATCTTGTACAAACAAATGC-3
+13854	FP 5-GCCCTAACCCCAGCCTTTGTTT-3<br>RP 5-GTATCGATCGTTCTGTATCAGTCTTTCC-3
Sat α	FP 5-ATCGAATGGAAATGAAAGGAGTCA-3<br>RP 5’-GACCATTGGATGATTGCAGTCA-3’
Gene desert chr.12	FP 5-GCTGTTACTTTTTACAGGGAGTTTTTA-3<br>RP 5-ATAAAGCAGGTAAAGGTCCATATTTC-3
HOXA11	FP 5-TTTCCACAGCCTTTGCAGGCG-3<br>RP 5-TTCAGGCTGCAAGAAGAAGCGGAG-3
PABPC1	FP 5-TGACACAGAGCCAGAAGTTGTAAAA-3<br>RP 5-CATGCATTGATACATCCTGCCTAA-3

Table 2. P-values of ChIP-qPCR signals for the different amplicons shown in Fig. 3A and B. The p-values refer to the comparison of the signals at day 5 with signal in native state or at day 3, day 10 or day 15.

H3K9me2 antibody

Amplicon	Native	Day3	Day10	Day 15
-4896	7.46E-03	8.67E-03	5.91E-02	1.24E-02
-890	1.70E-02	3.13E-03	5.00E-02	4.04E-03
-650	2.12E-02	4.36E-03	5.89E-01	5.08E-02
-189	7.77E-10	4.90E-08	4.74E-08	3.83E-09
+36	1.16E-03	3.31E-04	1.44E-01	1.28E-03
+205	3.22E-04	4.27E-04	2.92E-02	3.06E-04
+3529	9.34E-07	1.65E-07	1.52E-05	5.79E-07
+7275	1.93E-02	1.05E-02	7.35E-02	1.77E-02
+11266	4.64E-03	1.61E-03	1.16E-02	1.48E-03
+13854	1.38E-03	7.73E-04	2.48E-03	1.21E-03

H3K9me3 antibody

Amplicon	Native	Day3	Day10	Day 15
-4896	1.84E-04	1.73E-03	1.92E-04	2.81E-04
-890	2.46E-01	6.44E-01	3.64E-05	9.18E-06
-650	1.72E-03	8.03E-02	5.44E-03	9.87E-04
-189	8.85E-07	1.14E-05	1.53E-06	9.36E-07
+36	2.20E-03	8.19E-03	6.18E-04	5.49E-03
+205	6.29E-08	9.47E-07	2.46E-09	2.43E-08
+3529	4.01E-03	3.41E-03	3.28E-04	3.79E-05
+7275	1.79E-02	1.13E-02	1.80E-03	7.40E-04
+11266	4.05E-04	4.69E-03	4.95E-04	1.11E-04
+13854	8.81E-04	8.10E-03	9.57E-05	2.58E-05
